# The Mpox contextual data specification package: a data curation toolkit to support collaborative pathogen genomic surveillance

**DOI:** 10.1099/mgen.0.001614

**Published:** 2026-01-23

**Authors:** Emma J. Griffiths, Rhiannon Cameron, Charlotte Barclay, Nithu Sara John, Damion Dooley, Ivan Gill, Madeline Iseminger, Muhammad Zohaib Anwar, Mark E. Horsman, Keith MacKenzie, Natalie Prystajecky, John Tyson, Agatha Jassem, Tracy Lee, Rob Azana, Janet Fung, Michael Chan, Branco Cheung, Frankie Tsang, Dan Fornika, Jessica M. Caleta, Tara Newman, Kevin C. Yang, Shannon L. Russell, James E.A. Zlosnik, Linda Hoang, Natalie Knox, Andrea D. Tyler, Emily Haidl, Chanchal Yadav, Ana T. Duggan, Levon Kearney, Christopher Townend, Bernadette Cruz, Gary Van Domselaar, William W.L. Hsiao

**Affiliations:** 1 Centre for Infectious Disease Genomics and One Health (CIDGOH), Faculty of Health Sciences, Simon Fraser University, Burnaby, BC, Canada; 2 Public Health Ontario, Toronto, ON, Canada; 3 National Microbiology Laboratory, Public Health Agency of Canada, Winnipeg, MB, Canada; 4 Roy Romanow Provincial Laboratory, Regina, SK, Canada; 5 BCCDC Public Health Laboratory, Vancouver, BC, Canada

**Keywords:** contextual data, data management, genomic surveillance, harmonization, Mpox, Mpox virus (MPXV)

## Abstract

A sudden increase in the number of Mpox virus (MPXV) cases worldwide prompted the WHO to declare a Public Health Emergency of International Concern in 2022 and again in 2024. Public health genomic surveillance of MPXV in impacted areas is ongoing to inform national and international situational awareness, with a growing number of sequences available in public sequence repositories. Critical to genomic surveillance is well-curated and harmonized contextual data – the sample metadata, epidemiological and clinical data, lab results and method information that enables the interpretation of sequence data for public health responses and decision-making. Contextual data, however, is often unstructured or highly variable in formats, granularity and terminology. This variability usually requires a great deal of manual clean-up before it can be integrated and used for analysis, which can be laborious, time-consuming and error-prone. To facilitate harmonization of contextual data for genomic surveillance during the 2022 and 2024 epidemics, an MPXV contextual data specification was developed by the Centre for Infectious Disease Genomics and One Health (Simon Fraser University, Canada) in collaboration with several teams at Canada’s National Microbiology Lab [Public Health Agency of Canada (PHAC)] as well as provincial public health laboratories. The MPXV specification provides standardized ontology-based fields and terms for capturing information about MPXV samples and infections and prioritizes geo-temporal, data provenance and sampling strategy information for surveillance. The specification utilizes the same semantic framework used to develop other public health pathogen genomics data standards, thus demonstrating its adaptability for additional infectious diseases. The specification has been implemented as a template within an open-source application known as the DataHarmonizer, which provides curation, validation and data transformation features and functions. The MPXV specification is already being utilized in Canada and is freely available for international use. The MPXV specification adds to a growing library of interoperable, harmonized community consensus contextual data standards for public health pathogen genomics.

**Impact Statement**

Genomic surveillance of the Mpox virus (MPXV) is critical for understanding its evolution, transmission and for informing public health interventions. MPXV contextual data (metadata) is highly variable in public, and often private, repositories due to the lack/minimal use of data standards. This work describes an MPXV contextual data specification that provides ontology-based fields and terms, as well as standardized formats for harmonizing genomic contextual data across laboratories and systems. The standard is based on a common framework used to develop a growing library of interoperable specifications. The data standard offers solutions for a wide variety of data harmonization challenges which are showcased in a comprehensive series of worked examples. The specification was implemented in a data curation tool called the DataHarmonizer, which was used by provincial and federal Canadian public health laboratories as part of their data quality workflows during the 2022 MPXV epidemic. Harmonized data was used to distinguish importation events from local transmission, among other public health priorities. A subset of the fields is also implemented by the public repository Pathoplexus.

The use of the standard by provincial health authorities, a national reference lab and a public repository illustrates how standards can support the local-to-global data ecosystem. The standard is continually updated and freely available for international use.

## Data Summary

1. The Mpox specification package is freely and publicly available at https://github.com/cidgoh/MPox_Contextual_Data_Specification.
2. Harmonized Canadian Mpox contextual data can be found in Biosample records stored in NCBI BioProject PRJNA846794 (last accessed October 2024).
3. GenBank accessions for consensus sequences and additional worked examples are included in Supplementary Material (Table S1).

The authors confirm all supporting data, code and protocols have been provided within the article or through supplementary data files.

## Introduction

On 14 August 2024, the World Health Organization (WHO) declared that the current Mpox epidemic was a Public Health Emergency of International Concern [1]. Mpox is a disease caused by the monkeypox virus (MPXV), a virus from the *Orthopoxvirus* genus in the family Poxviridae – the same family of viruses that causes smallpox [2]. Mpox symptoms are similar to smallpox symptoms, but milder, and include a rash that can look like pimples or blisters, fever, swollen lymph nodes and body aches [2]. The Mpox case fatality rate varies depending on the viral clade and other factors, but generally ranges from 0% to 11% in outbreaks in endemic areas, with mortality mostly affecting young children and immunocompromised individuals [2,3]. MPXV infection is normally caused by spill-over events to humans from animals such as rodents, squirrels and non-human primates [2]. The virus can also be transmitted person-to-person by close contact with lesions, body fluids, respiratory droplets and contaminated materials, especially with more recent variants that have evolved for more efficient transmission [2]. Mpox was previously declared to be a Public Health Emergency of International Concern on 23 July 2022 [4]. At the time of writing, the WHO reports over 123 countries have reported Mpox between January 2022 and August 2024, with over 137,892 laboratory-confirmed cases and over 317 deaths among confirmed cases [5].

Case counts and epidemiological patterns suggest that the current global outbreak is sustained by human-to-human transmission [6,7]. Genomic surveillance of MPXV is ongoing, with a growing number of sequences available in public sequence repositories such as the International Nucleotide Sequence Database Collaboration (INSDC), the Global Initiative on Sharing Avian Influenza Data (GISAID) EpiPox database, and Pathoplexus [8,9, 10, 11]. MPXV genomic data has provided insights into Mpox phylodynamics and transmission dynamics during both epidemics. The 2022 epidemic was largely driven by MPXV clade II, which was associated with more mild disease severity [12]. The current (2024/2025) epidemic appears to be largely driven by MPXV clade Ib, which has been associated with higher mortality (mainly in children) and possibly increased transmissibility [13,14]. Well-harmonized contextual data is critical to making informed decisions and has been essential for global MPXV surveillance and other genomic surveillance programmes Mussen et al 2022 [15,25].

Contextual data consists of sample metadata, epidemiological and clinical data, lab testing results and methodological information that provides essential context for the interpretation, integration and analysis of sequence data. Contextual data is often generated by different agencies and laboratories using different case report forms (public health questionnaires documenting details of a person’s illness), as well as different information management systems which collect and structure different types of data at different levels of granularity, in different formats, using heterogeneous semantics (i.e. fields and terms). The variability in the contextual data usually requires manual clean-up before it can be integrated and used for analysis, which can be laborious, time-consuming and error-prone [26]. Contextual data is often structured using organization-specific data dictionaries, minimum information checklists or free text [27,30]. Using these mechanisms for harmonizing contextual data can create challenges for the interoperability of datasets, as there is rarely a ‘one-size-fits-all’ terminology or nomenclature system.

Ontologies are sets of hierarchical controlled vocabulary, in which terms are linked by logical relationships [31,32]. The meanings of terms are meant to be universal rather than institution- or project-specific and are disambiguated using universal identifiers [Internationalized Resource Identifiers (IRIs)] [31,32]. With the emphasis on common meaning rather than relying solely on term labels, ontologies incorporate synonyms and database cross-references [31,32]. Ontologies also provide the ability to relate entities via axioms and hierarchical groupings, which better enables standardized classification schemes and the construction of knowledge graphs for more complex queries and the application of artificial intelligence approaches. To facilitate MPXV contextual data harmonization and integration across datasets, laboratories and systems, an ontology-based MPXV contextual data specification was developed by the Centre for Infectious Disease Genomics and One Health (CIDGOH) at Simon Fraser University, in collaboration with several teams at Canada’s National Microbiology Lab (Public Health Agency of Canada) and provincial public health laboratories (Canadian Public Health Laboratory Network). The specification was built using an interoperable, modular, International Organization for Standardization (ISO)-based framework that has been re-used for other genomic surveillance initiatives (e.g. Severe acute respiratory syndrome coronavirus 2 (SARS-CoV-2), One Health AMR and Wastewater) [18,19, 33]. The standard was also aligned with technical guidance documentation in preparation by the WHO’s International Pathogen Surveillance Network (IPSN).

The implementation of contextual data standards and harmonization tools has been shown to enable faster and more efficient data harmonization and integration during routine operations as well as public health emergencies [18,20,34]. One such data management tool is called the DataHarmonizer, which enables data entry and validation against a computer- and human-readable data specification. This tool also enables automated data transformation into submission-ready formats required for institutional-specific databases and public repositories including the Global Initiative on Sharing All Influenza Data (GISAID) database and the National Center for Biotechnology Information (NCBI) [35]. This automated transformation helps to reduce errors and the burden of manual reformatting. The DataHarmonizer is a browser-based, template-driven spreadsheet application which offers various data collection templates based on consensus data standards and provides curation/validation tools and reference guides which enable data providers to securely work with their contextual data offline, avoiding the public health security issues that arise with online services [35]. The DataHarmonizer was developed to address data sharing needs that arose during the COVID-19 pandemic and is currently used by members of the Canadian Public Health Laboratory Network (CPHLN) to harmonize various national genomic surveillance data. The MPXV data specification was implemented as a data collection template within the DataHarmonizer and was made available to all laboratories at the provincial, territorial and federal levels during the 2022 Mpox epidemic. As such, the data specification was used for collaborative data collection and surveillance according to national public health priorities, and harmonized data using the specification package has been shared publicly.

In response to the 2024 MPXV epidemic, the data specification was internationalized and provided as an additional template within the DataHarmonizer (https://github.com/cidgoh/MPox_Contextual_Data_Specification). The specification package includes the templates, as well as a curation protocol and field and term reference guides to provide better clarity in the meaning of vocabulary. Development of the MPXV specification – and the harmonization challenges that it helps to solve – are described in this work. The standard and tools are available for immediate international use.

## Theory and implementation

### Data needs assessment

Data needs for MPXV surveillance were assessed via consultations with public health partners as well as review of case report forms and technical genomic surveillance guidance documents (e.g. WHO, PHAC and IPSN), public repository requirements, genomic surveillance literature, Genomics Standards Consortium minimum information checklists and existing ontology-based pathogen surveillance specifications. The scope of the specification included different lineages of MPXV; different samples and hosts; associated clinical, epidemiological and laboratory data; and tiled amplicon and targeted (i.e. bait capture) and shotgun metagenomic sequencing approaches. It should be noted that the international data needs were broader in scope than the identified Canadian needs, which are focused on clinical samples. Vocabulary, in both cases, was needed to capture different types of identifiers and to describe diverse sample types and sample processing methods, sampling strategies, host demographics, symptoms, risk factors, vaccination, treatment, exposures, cases of re-infection, wet lab and bioinformatic methods (e.g. extraction, library preparation, amplicon sequencing schemes, enrichment, sequence processing and mapping to reference databases, etc), diagnostic test results (e.g. PCR Ct values), data provenance and contribution acknowledgement. The assessment also revealed additional differences between Canadian and international data collection data needs, e.g. additional Canadian-specific sample identifiers and linkage annotations (e.g. ‘Related specimen primary ID’), and there were often a smaller number of possible inputs for many Canadian data elements compared with international data collection scenarios. The data needs assessment also highlighted common harmonization challenges which included identifier tracking and mapping across databases and platforms; standardized structure of sample, host, clinical and exposure information; and capture of nuanced experimental design, wet lab and bioinformatic methods. After data needs were assessed, vocabulary was compiled to reflect those needs.

### Specification structure and standardization

Standardized fields and terms were sourced from Open Biological and Biomedical Ontology (OBO) Foundry ontologies and where vocabulary was missing, new terms were developed and submitted to appropriate resources within the Foundry. The OBO Foundry is a semantics community of practice that consists of an expanding group of scientists committed to developing interoperable ontologies. The OBO Foundry prescribes a set of principles and practices, which include the use of common formal languages and syntax. Terms can be searched in different registries and look-up services, e.g. EBI Ontology Look-up Service (https://www.ebi.ac.uk/ols4/). In total, 24 different OBO Foundry ontologies were utilized in developing the specification (Table 1), enhancing interoperability across different datasets due to the common architecture of the different domain ontologies used. Ontology identifiers were included with vocabulary within the specification rather than relying solely on term labels in order to improve clarity of meaning and to provide a mechanism to support multilingual translations. While the Mpox specification is currently provided to users in English, it is possible to develop a French language equivalent. Multilingual data transformations are especially important in the Canadian context where public health agencies must often operate in both official languages (English and French). For example, the GENEPIO prefix in the ontology identifier ‘Swab (GENEPIO:0100027)’ represents the Genomic Epidemiology Ontology source ontology and the ‘0100027’ numerical value identifies the term Swab within GenEpiO. It should be noted that currently, OBO Foundry ontologies are primarily available in English; however, there are different efforts underway to provide multilingual translations as needed. In some cases, French translations of terms were available as they had been previously provided by the Public Health Agency of Canada (e.g. ‘Écouvillon’ for ‘Swab’) during other work. Including ontology identifiers when storing or sharing data better enables organizations to use whatever vocabulary they prefer (e.g. different synonyms or translations) while enabling systems to operate using a common language (based on identifiers). Fields and terms were also structured using best practices derived from different communities of practices (e.g. FAIR data, software development and semantics) for improving machine-readability, longevity and discoverability. These principles and practices – highlighted in previous standard papers [33] – help prepare data for more complex queries and artificial intelligence analyses (e.g. machine learning) and will be discussed more fully in upcoming manuscripts (Cameron *et al*., in preparation; Barclay *et al.*, in preparation).

**Table 1.** List of 24 OBO Foundry ontologies used to source the Mpox specification’s standardized fields and terms

Ontology	Domain	URL
BTO	Biological tissues	https://obofoundry.org/ontology/bto.html
ChEBI	Chemicals	https://obofoundry.org/ontology/chebi.html
DOID	Human disease	https://obofoundry.org/ontology/doid.html
ECTO	Environmental exposure	https://obofoundry.org/ontology/ecto.html
EFO	Experimental factor	https://www.ebi.ac.uk/efo/
EnvO	Environments	https://obofoundry.org/ontology/envo.html
FMA	Human anatomy	https://obofoundry.org/ontology/fma.html
FOODON	Food	https://obofoundry.org/ontology/foodon.html
GAZ	Geography	https://obofoundry.org/ontology/gaz.html
GENEPIO	Genomic epidemiology	https://obofoundry.org/ontology/genepio.html
GSSO	Gender, sex, sexual orientation	https://obofoundry.org/ontology/gsso.html
HP	Human phenotypes	https://obofoundry.org/ontology/hp.html
IDO	Infectious disease	https://obofoundry.org/ontology/ido.html
MMO	Measurement methods	https://obofoundry.org/ontology/mmo.html
MONDO	Multi-organism diseases	https://obofoundry.org/ontology/mondo.html
MP	Mammalian phenotypes	https://obofoundry.org/ontology/mp.html
NCBITaxon	Disease taxonomy	https://obofoundry.org/ontology/ncbitaxon.html
NCIT	National Cancer Institute Thesaurus	https://obofoundry.org/ontology/ncit.html
OBI	Assays, devices, experiments, etc.	https://obofoundry.org/ontology/obi.html
OMRSE	Social entities related to healthcare	https://obofoundry.org/ontology/omrse.html
PCO	Populations and communities	https://obofoundry.org/ontology/pco.html
SYMP	Symptoms	https://obofoundry.org/ontology/symp.html
TRANS	Disease transmission	https://obofoundry.org/ontology/trans.html
UBERON	Multi-organism anatomy	https://obofoundry.org/ontology/uberon.html
UO	Units	https://obofoundry.org/ontology/uo.html

Vocabulary was grouped within different thematic modules, and the specification was structured according to a modular, ISO-based framework described elsewhere [19]. Modules included database identifiers, sample collection and processing, host information, host vaccination information, host exposure information, host reinfection information, sequencing, bioinformatics and QC metrics, pathogen diagnostic testing and contributor acknowledgement. Two subsets of vocabulary were created − one representing international data collection needs, the other reflecting Canadian-specific needs. The international subset contained 166 fields and the Canadian subset contained 141 fields. Picklists of values were provided for many of the fields in both subsets, though the composition of the picklists sometimes varied according to the range of possible inputs (e.g. the range of Canadian inputs was often smaller). It should be noted that while there are a large number of fields provided, only 21 are considered to be ‘required’ in the international subset. These fields have been prioritized for capturing data provenance, methods, sampling strategies and geo-temporal information about how samples were collected, based on previous experience developing international data standards. These fields have been shown to be the most critical for genomic surveillance in establishing when and where a pathogen is present and are also considered less sensitive than other epidemiological data with privacy and identifiability concerns. The required set of fields is presented in Table 2. The Canadian implementation also has an increased number of required fields (total of 33) due to existing data sharing agreements between jurisdictions. A comparison of international and Canadian required fields is presented in Table 3.

**Table 2.** The Mpox required fields represent a ‘reusable core’ that can be applied to additional pathogen standards These fields can also facilitate data sharing and governance by establishing consistent, common elements for memorandums of understanding across initiatives and partners.

Field	Definition	Ontology ID
specimen collector sample ID	The user-defined name for the sample	GENEPIO:0001123
sample collected by	The name of the agency that collected the original sample	GENEPIO:0001153
sequenced by	The name of the agency that generated the sequence	GENEPIO:0100416
sequence submitted by	The name of the agency that submitted the sequence to a database	GENEPIO:0001159
sample collection date	The date on which the sample was collected	GENEPIO:0001174
geo_loc_name (country)	The country where the sample was collected	GENEPIO:0001181
geo_loc_name (state/province/territory)	The state/province/territory where the sample was collected	GENEPIO:0001185
organism	Taxonomic name of the organism	GENEPIO:0001191
isolate	Identifier of the specific isolate	GENEPIO:0001195
purpose of sampling	The reason that the sample was collected	GENEPIO:0001198
purpose of sampling details	The description of why the sample was collected, providing specific details	GENEPIO:0001200
host (scientific name)	The taxonomic, or scientific name of the host	GENEPIO:0001387
host disease	The name of the disease experienced by the host	GENEPIO:0001391
purpose of sequencing	The reason that the sample was sequenced	GENEPIO:0001445
purpose of sequencing details	The description of why the sample was sequenced providing specific details	GENEPIO:0001446
sequencing instrument	The model of the sequencing instrument used	GENEPIO:0001452
consensus sequence software name	The name of software used to generate the consensus sequence.	GENEPIO:0001463
consensus sequence software version	The version of the software used to generate the consensus sequence	GENEPIO:0001469
raw sequence data processing method	The names of the software and version number used for raw data processing such as removing barcodes, adapter trimming, filtering etc.	GENEPIO:0001458
dehosting method	The method used to remove host sequence data from the pathogen sequence data	GENEPIO:0001459
bioinformatics protocol	A description of the overall bioinformatics strategy used	GENEPIO:0001489

**Table 3.** Comparison of required fields between the ‘Mpox_international’ and Canadian Mpox templates, highlighting customization specific to different jurisdictions A total of 21 fields are considered ‘required’ for MPXV genomic surveillance in the international specification, while a total of 33 fields are required in the Canadian implementation. The Canadian implementation minimal set has been customized to include two additional fields for NML LIMS data management purposes and ten additional fields pertaining to sample type and host demographics that are only ‘recommended’ in the international specification. The additional requirements in the Canadian specification are permissible due to standing data sharing agreements between public health jurisdictions.

International specification	Canadian customized specification
**specimen collector sample ID**	**specimen collector sample ID**
**sample collected by**	**sample collected by**
**sequenced by**	**sequenced by**
**sequence submitted by**	**sequence submitted by**
**sample collection date**	**sample collection date**
***No international equivalent***	**sample collection date precision**
**geo_loc_name (country**)	**geo_loc_name (country**)
**geo_loc_name (state/province/territory**)	**geo_loc_name (state/province/territory**)
**organism**	**organism**
**isolate**	**isolate**
**purpose of sampling**	**purpose of sampling**
**purpose of sampling details**	**purpose of sampling details**
***No international equivalent***	**NML submitted specimen type**
***‘Recommended’ in international specification***	**anatomical material**
***‘Recommended’ in international specification***	**anatomical part**
***‘Recommended’ in international specification***	**body product**
***‘Recommended’ in international specification***	**collection device**
***‘Recommended’ in international specification***	**collection method**
**host (scientific name**)	**host (scientific name**)
**host disease**	**host disease**
***‘Recommended’ in international specification***	**host age**
***‘Recommended’ in international specification***	**host age unit**
***‘Recommended’ in international specification***	**host age bin**
***‘Recommended’ in international specification***	**host gender**
**purpose of sequencing**	**purpose of sequencing**
**purpose of sequencing details**	**purpose of sequencing details**
***‘Recommended’ in international specification***	**sequencing date**
**sequencing instrument**	**sequencing instrument**
**raw sequence data processing method**	**raw sequence data processing method**
**dehosting method**	**dehosting method**
**consensus sequence software name**	**consensus sequence software name**
**consensus sequence software version**	**consensus sequence software version**
**bioinformatics protocol**	**bioinformatics protocol**

Owing to the range of sample type fields included in the international subset, it is suitable for One Health investigations and studies and includes standardized sample descriptors for animal hosts (e.g. rodents) and environmental sources (e.g. bed linen, clothing and wastewater), in addition to clinical samples. Both sets of vocabulary prescribe formatting for dates as per ISO 8601 (YYYY-MM-DD) and provide fields to track different identifiers and accessions for the same samples across multiple public repositories (e.g. INSDC, GISAID and Pathoplexus). A set of standardized null values sourced from the INSDC repository documentation (i.e. missing, not applicable, not provided, not collected and restricted access) were also included in picklists. Maximum and minimum value ranges, data types and other patterns were also included in these specifications which were implemented as templates within the data harmonization tool called the DataHarmonizer. Template development is described in Tooling and support materials.

### Framework generalizability and international use

The modules of the semantic framework used to structure the MPXV specification consist of fields and terms thematically grouped together to form a unit. These ‘plug and play’ modules can be reused to create a specification for almost any target or pathogen. Modules can also be enriched/depleted with fields/terms based on particular data needs. Reuse of the framework helps to create consistency between specifications, as well as interoperability between datasets and systems that implement them. Consistency in data capture helps build curation capacity as practitioners are familiar with vocabulary, formats and requirements, and this consistency also helps streamline data management by reducing redundancy across pathogen areas.

The semantic framework has been used previously to create both Canadian and international data standards for different pathogens and surveillance approaches. For example, it was used by the Public Health Alliance for Genomic Epidemiology (PHA4GE; https://pha4ge.org/) to create a data specification for SARS-CoV-2 during the COVID-19 pandemic, as well as a wastewater genomic surveillance specification [18,33]. PHA4GE aims to improve the reproducibility, interoperability, portability and capacity for public health bioinformatics around the world. The PHA4GE Data Structures Working Group has adopted this framework for standards development and is currently using it for the development of other specifications (e.g. highly pathogenic avian influenza, malaria, cholera and sepsis).

**Fig. 1.**
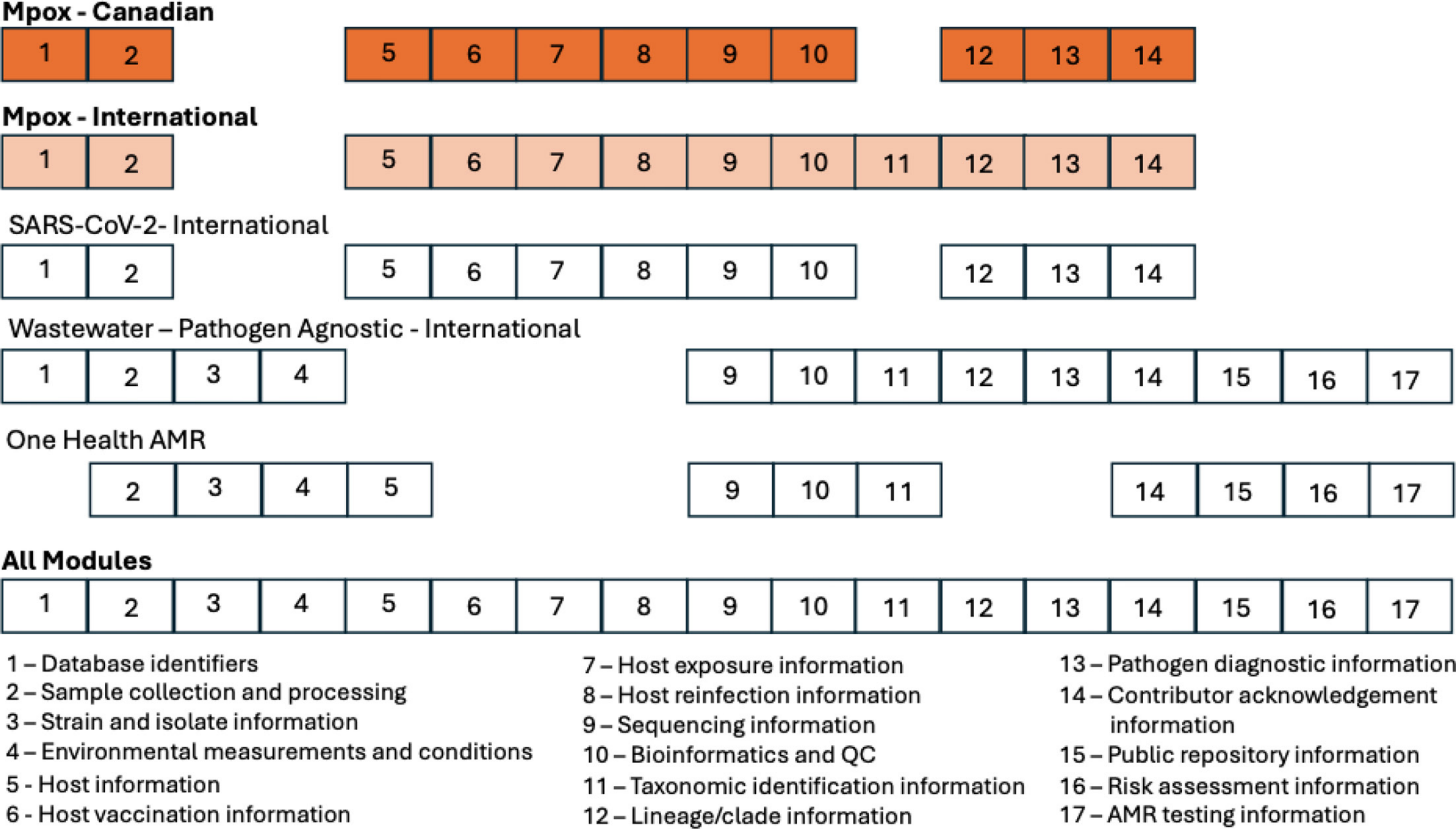
Comparison of modules used across the Canadian and International Mpox, as well as other interoperable specifications – including Canada’s One Health AMR standard and PHA4GE’s SARS-CoV-2 and wastewater specifications built using the same framework. Many modules are reused across standards (e.g. database identifiers, sample collection and processing, sequencing information, bioinformatics and QC, pathogen diagnostic testing and contributor acknowledgements). Specific data needs for different surveillance approaches and programmes are addressed by new (reusable) modules. For example, the Mpox standard contains modules for host-associated information (e.g. demographics, exposures, clinical information, vaccination and treatment information, etc.) while the wastewater standard contains a module for physico-chemical measurements and sampling conditions, and the One Health AMR standard contains modules for Risk assessment and AMR phenotypic testing. Labs using clinical samples for genomic surveillance are recommended to use the Mpox specification for harmonization and future-proofing data, while labs using wastewater approaches to Mpox surveillance are recommended to use the Wastewater specification. The interoperability created using the ontology and modular approaches better enables Mpox data to be integrated across clinical and environmental detection strategies compared to datasets structured using incompatible standards or no standards at all.

A subset of the wastewater specification was used to create new Biosample packages at both NCBI and the European Nucleotide Archive (ENA), facilitating international data sharing (https://www.ncbi.nlm.nih.gov/biosample/docs/packages/PHA4GE.wwsurv.1.0/). The framework was also used to create a data standard for One Health antimicrobial resistance, developed as part of a Canadian inter-agency Genomics Research and Development Initiative for One Health Antimicrobial Resistance surveillance [19]. The specification is being used to harmonize and share data in an international collaboration between Canada and the UK [19]. A subset of the GRDI-AMR specification was used to structure One Health data in a national study involving various labs and agencies across Uganda [19]. Picklists were updated to reflect sample types, sampling strategies, locations and food types specific to the Ugandan context. A subset of the GRDI specification is also being included as part of gold standard benchmark datasets in the JPIAMR-funded B2B2BAMRDx initiative [19]. A comparison of modules across different standards is illustrated in Fig. 1. This customization and reuse of the framework demonstrates its adaptability and suggests its potential use for additional infectious diseases.

### Tooling and support materials

**Fig. 2.**
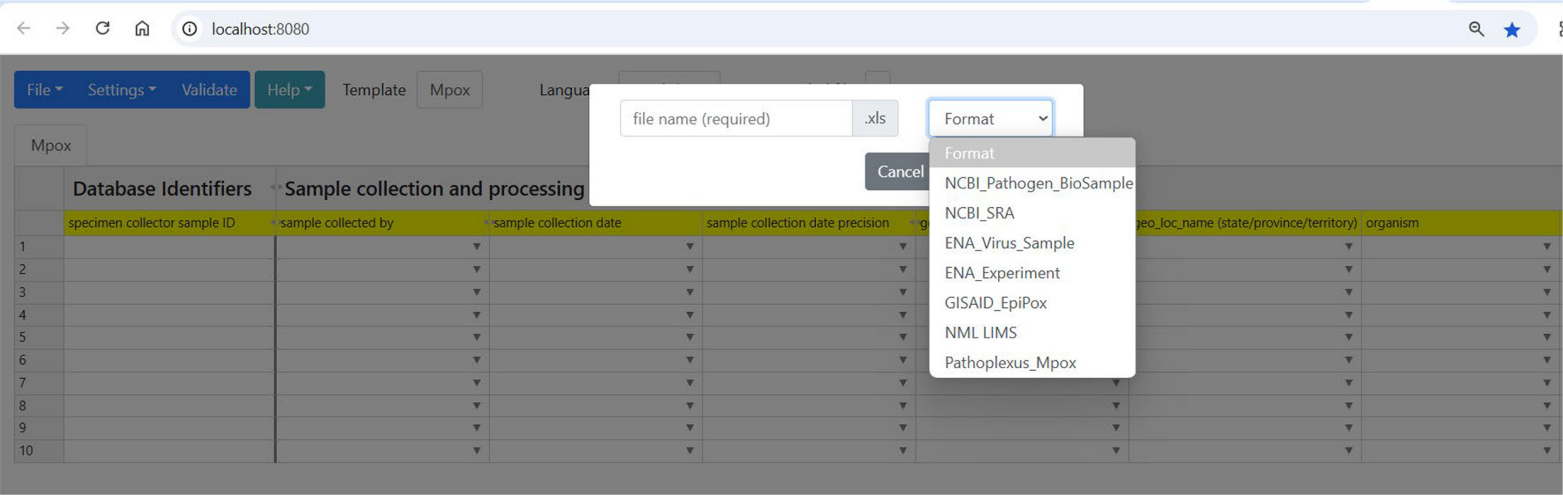
Data standards and tooling better enable interoperability and data sharing across repositories and public health surveillance databases. The DataHarmonizer’s export functionality automates contextual data transformations based on mapping and integrated exchange formats. Entered data can be reformatted and exported as submission-ready (or near submission-ready) spreadsheets according to NCBI’s Pathogen BioSample, NCBI’s SRA, ENA’s Virus Sample checklist (ERC000033), ENA’s Experimental metadata, Pathoplexus, GISAID EpiPox and PHAC-NML’s genomic database requirements. Export formats are in .xls format.

While data standards provide a means for structuring and harmonizing contextual data, standards must be implemented in data management tools to enable them to be put into practice in public health laboratories. The DataHarmonizer is a spreadsheet-based text editor application to support data curation and validation, as well as automated transformations to better enable data sharing with public repositories. The DataHarmonizer can be downloaded locally and does not require an internet connection to operate. Specifications are implemented as templates containing standardized fields, terms and formats, which can be populated with data by public health practitioners. The Mpox specification is implemented as two distinct templates named ‘Mpox_international’ and ‘Mpox’ (representing the Canadian data collection template). The DataHarmonizer enables users to enter text directly or upload data as an xlsx, tsv or csv file; it provides features for curation (e.g. Fill Column; Jump To; show just required, required+recommended, all fields; and show invalid fields), as well as validation. To ease the burden of reformatting of data for public repository submission, the specifications were mapped to downstream repository data elements and exchange formats were developed. The exchange formats were implemented as DataHarmonizer exports – enabling users to harmonize their contextual data according to the data standard and then perform automated transformations to generate submission-ready data via the Export function (Fig. 2). Exports are available for GISAID EpiPox, NCBI’s Pathogen BioSample package, NCBI’s SRA package, ENA’s Virus Biosample package (ERC000033), ENA’s Experimental Data package, Pathoplexus submission requirements and the Public Health Agency of Canada’s national genomics database (NML LIMS). Templates are updated as needed and version controlled. The Mpox and Mpox_international templates are available at https://github.com/cidgoh/MPox_Contextual_Data_Specification.

DataHarmonizer template development has been described elsewhere, but briefly, pathogen genomic schemas in the DataHarmonizer are built to conform to the Linked Data Modeling Language (LinkML; https://linkml.io/) for schemas and data dictionaries. The LinkML Generator framework generates downstream artefacts, including JSON-Schema (semantic web-ready, and enables the use of JSON validators), ShEx, RDF, OWL, GraphQL and SQL DDL, which help support the integration of specifications into downstream applications. The LinkML framework also automates publishing schema documentation using mkdocs and assists in publishing schema artefacts using w3id.org.

A LinkML schema may contain classes, slots, enums, types and settings. A LinkML class is used to hold a spreadsheet template specification. It can define its own slots (fields) or draw from a common pool of slots that the schema provides for any template to use. A LinkML slot holds a column field specification, so it controls the data entry and validation for a spreadsheet column’s cells. A LinkML enum holds a flat or hierarchical list of categorical picklist values that can be used by one or more slots. A LinkML type often corresponds to a database field data type and is referenced in a slot’s range of acceptable values. Templates sharing common schema elements enable updates to be propagated more easily across templates. Template vocabularies are developed and tracked using Google spreadsheets and DataHarmonizer templates are stored as JSON files and used to dynamically generate the application interface and its functionality using the Handsontable (9.0.2) JavaScript spreadsheet application. Open-source code and further technical details are available at https://github.com/cidgoh/DataHarmonizer/wiki/DataHarmonizer-Templates

‘Canadian’ and ‘International’ Mpox DataHarmonizer templates were constructed based on a common LinkML schema.

The Canadian Mpox template was initially released in June 2022 when it was used for harmonizing Canadian public health genomic contextual data during the 2022 epidemic and was subsequently refined based on user feedback. Vocabulary was added as needed and requested. The international Mpox template was also initially released in July 2022, with an expanded set of epidemiology and sample type fields compared to the Canadian template. The international template was reviewed after the 2024 WHO PHEIC declaration and updated. Bug fixes and additional features (e.g. reporting DataHarmonizer and template versions in the provenance field) were included in subsequent releases. Test dataset files are included with each new release as part of the tool’s quality management practices.

Recognizing that data needs evolve over time, the Pathogen Genomics Package repository offers a standardized New Term Request (NTR) form that enables the community to make term requests for missing vocabulary. Requests require that a user includes the name of the requested term, any synonyms or alternative labels, a definition and its source, and contact information for follow-up by a data curator. The curator will identify an existing term match if possible but will otherwise identify the appropriate parent ontology and hierarchical placement for the term and reach out to different ontology developers for inclusion of the new vocabulary. Updates to the specification will occur periodically. These processes are intended to encourage communication between standard developers, data providers and data users, as well as encouraging community development of data management tools. The NTR is available as a submission form in GitHub issue tracker.

The Mpox specification is version controlled on GitHub. All edits and updates are tracked in release notes. Versioning is captured in the format of x.y.z, where

x=Field level changes

y=Term value/ID level changes

z=Definition, guidance, example, formatting, or other uncategorized changes.

Ongoing user feedback and error reporting is encouraged via the Mpox specification’s GitHub repository issue tracker (https://github.com/cidgoh/MPox_Contextual_Data_Specification/issues). The use of the GitHub issue tracker is helpful for transparency and for long-term documentation of community needs.

To support data curation, the Mpox specification package also provides users with field and term reference guides (containing definitions, guidance for populating fields and examples). A curation protocol (SOP) is also available which provides DataHarmonizer operating instructions, additional curation guidance for new users, as well as different privacy, ethical and practical considerations for capturing and sharing data. A summary of resources included in the Mpox specification package is provided in Table 4.

**Table 4.** List of resources in the Mpox contextual data specification package The package includes the data standard encoded as LinkML, DataHarmonizer templates to implement the standard in public health settings, field and term reference guides providing definitions and additional guidance, a curation protocol including ethical and privacy considerations, a mapping file from the standard to public repository requirements and a new term request form to enable the community to communicate ongoing data needs to standard and software developers.

Resource	Description	Link
Collection templates and controlled vocabulary pick lists in the DataHarmonizer	Spreadsheet-based collection forms containing different fields (identifiers and accessions, sample collection and processing, host information, host exposure, vaccination and reinfection information, lineage/clade information, sequencing, bioinformatics and quality control metrics, diagnostic testing information and author acknowledgements); fields are colour-coded to indicate required, recommended or optional status; many fields offer pick lists of controlled vocabulary	https://github.com/cidgoh/pathogen-genomics-package/releases
Field and term reference guides	Step-by-step instructions for using the collection template are provided in an SOP; ethical, practical and privacy considerations are also discussed; examples and instructions for structuring sample descriptions are also discussed	https://github.com/cidgoh/MPox_Contextual_Data_Specification/tree/main/Reference%20Guide
Mapping file of Mpox data specification fields to public repository requirements	Fields are mapped to NCBI Pathogen BioSample package and GISAID EpiPox submission requirements; mappings are available in the Field Reference guide tab	N/A
New term request form	N/A	https://github.com/cidgoh/MPox_Contextual_Data_Specification/issues/new/choose

N/A, not applicable.

### Testing and implementation

To ensure the specification was fit-for-purpose, the specification was tested using provincial datasets submitted to the NML. The development team took note of fields and terms that were poorly implemented due to lack of appropriate instruction or inappropriate/missing vocabulary, and incorporated feedback received in debriefing sessions. Reported benefits of the specification included easier integration of provincial data into the national genomics database and reduced error rates. Ongoing challenges included the need for manual curation from original data sources (i.e. spreadsheets and lab information management systems) into the specification format. The use of large language models to facilitate automated transformations from native data sources is now being investigated.

**Fig. 3.**
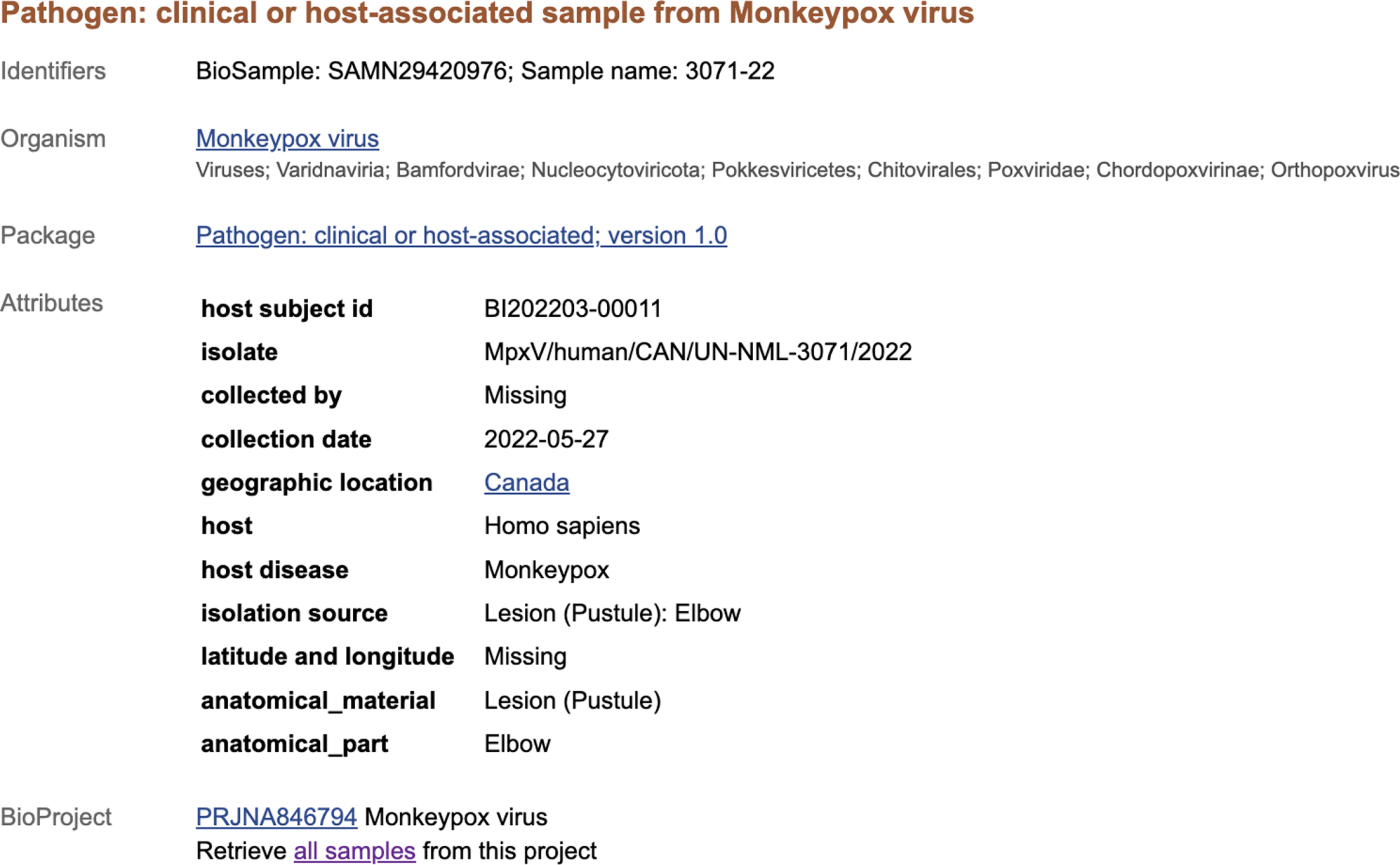
Example of a Canadian implementation of the Mpox specification in publicly available data. The NCBI BioSample record submitted by the Public Health Agency of Canada uses the fields of NCBI’s pathogen package combined with standardized terms from the Mpox specification. This structured data better enables integration across datasets compared to free text.

**Fig. 4.**
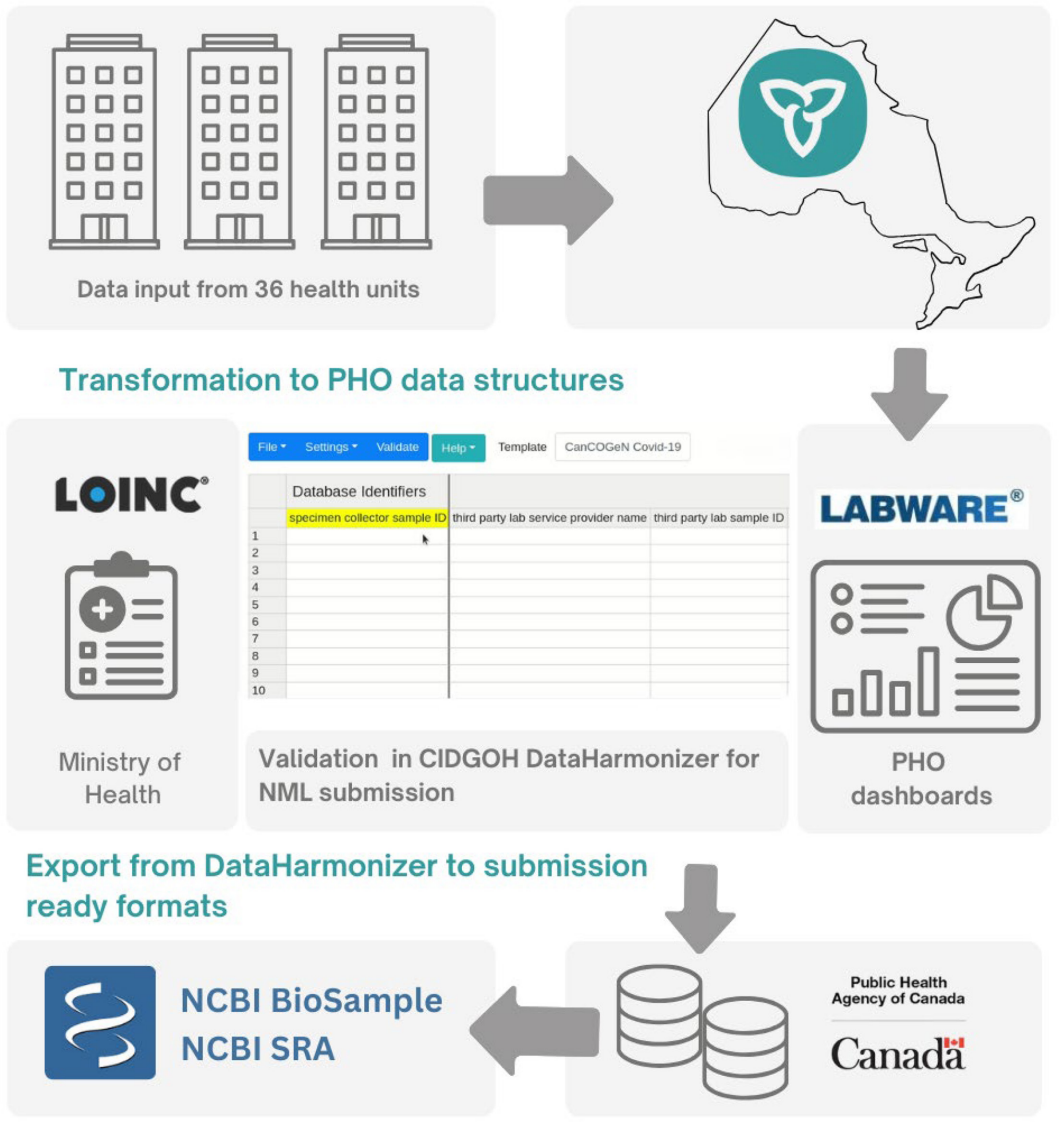
Case study illustrating how the Mpox standard fits into data capture and sharing processes in the Canadian Province of Ontario. The country of Canada consists of 10 provinces and three territories that all interact with the federal government. Canada has a federated health system, i.e. health falls under provincial/territorial jurisdiction, with cross-border health issues falling under federal jurisdiction. Each province/territory has its own public health authority, which works with local health units to coordinate surveillance and service delivery. Canada’s federal public health authority is called the Public Health Agency of Canada, which works with provincial labs to support national health priorities. Local health units, provincial laboratories and federal agencies all have different data systems. In Ontario, the provincial public health authority – Public Health Ontario (PHO) – collaborates with 36 local public health units. PHO stores different types of data in different systems. During the Mpox outbreak, PHO received samples for sequencing through requisitions submitted by authorized healthcare providers. These were logged and processed in LabWare, where test results were generated and reported to the provider, the local public health unit (PHU) and the Ministry of Health via the Ontario Laboratories Information System (OLIS). Genomic analyses and associated contextual data were compiled from LabWare and other sources. Data from these sources were formatted in accordance with the DataHarmonizer inputs and harmonized before submission to PHAC’s National Microbiology Laboratory (NML) and the National Genomics Database. The DataHarmonizer has the added capacity to output in NCBI BioSample and Sequence Read Archive (SRA) formats for international sharing. The figure demonstrates the fluidity of data in the Ontario health system and beyond. While the Mpox specification can provide harmonization solutions across some systems, the data is part of a more complex ecosystem.

Canadian data harmonized according to the Mpox specification was shared publicly and can be found in NCBI BioProject PRJNA846794. As of 29 September 2022, Canada has publicly shared 138 MPXV sequences using the Mpox data standard, anonymized for geo-location due to the low number of samples and to prevent re-identification. An example of standardized data from the BioProject is presented in Fig. 3. An example of how the Mpox specification was used in the context of the complexity of Canadian health data systems and beyond is provided in Fig. 4.

While plans for testing the international template are underway, many of the fields from this specification have been implemented by Pathoplexus – a new, open-source international data repository dedicated to the efficient sharing of human viral pathogen genomic data fostering global collaboration and public health response (https://pathoplexus.org/docs/concepts/metadataformat). At the time of writing, Pathoplexus hosts the largest number of publicly shared Mpox sequences of any global database (4,654 records and associated sequences). Furthermore, data from a 2025 MPXV outbreak in Sierra Leone was tracked and managed through the Pathoplexus platform using parts of the Mpox data standard (https://virological.org/t/genomic-epidemiology-of-mpox-virus-in-sierra-leone/995).

### Harmonization solutions

The Mpox specification offers a wide variety of standardized fields and terms aimed at improved capture of provenance information to better document chains of custody, well-structured sample descriptions for clinical and One Health samples, detailed experimental design as well as different types of laboratory, sequencing and bioinformatics methods. A variety of scenarios were selected to highlight contextual data standardization using these standards in diverse situations. Partial contextual data records are provided below to illustrate the use of different data provenance and identifier tracking, sample collection and processing, experimental design and methodology fields and picklist terms. Simulated data has been used in the worked examples to protect privacy. Additional worked examples highlighting identifier tracking, longitudinal sampling, vaccination, replicate tracking, PCR test methodology and results and taxonomy are provided in Supplementary Material.

#### Data traceability

Generating genomic sequence data from samples and using them in analyses can involve different partners and laboratories that may be responsible for different processes. Tracking the roles and contributions of different partners is not only good practice for auditability, articulating data traceability and maintaining contact information for follow-up, but is also good ethical practice for equitable benefit sharing. Furthermore, as samples, isolates and sequences transfer between labs and partners, they may be assigned different identifiers which may create confusion and errors if improperly tracked. Samples and any derived entities or data may also need to be associated with hosts, cases and different methodologies. To facilitate attribution, identifier tracking and establishing chains of custody, the Mpox specification provides a variety of different provenance fields. Scenarios and worked examples highlighting how different types of provenance fields can be used are provided below. See Supplementary Material for additional examples.

**Worked examples**

Scenario 1: An Mpox sample was collected by Chinua Tesfaye at the Ethiopian Public Health Institute (contact email: c.tesfaya@ephi.et), which was then sequenced by the Ethiopian National Reference Laboratory (contact: Dr Liya Aweke, aweke@enrl.et). The resulting sequence and associated contextual data was also submitted to a public repository by Dr Aweke. A partial contextual data record for the sample highlighting different attribution fields is provided below.

**sample_collected_by:** Ethiopian Public Health Institute

**sample_collector_contact_name:** Chinua Tesfaye

**sample_collector_contact_email:** c.tesfaya@ephi.et

**sequenced_by:** Ethiopian National Reference Laboratory

**sequenced_by_contact_name:** Liya Aweke

**sequenced_by_contact_email:** aweke@enrl.et

**sequence_submitted_by:** Liya Aweke

**sequence_submitter_contact_email**: aweke@enrl.et

#### Sample collection and processing

Information about samples often pertains to what was collected and from where, as well as how, when and why a particular sample was collected. Clinical Mpox samples can be collected from a wide array of different anatomical parts (structures) and may consist of different anatomical materials (substances) and body products (substances that are excreted/secreted). Samples may be collected using particular methods and devices. One Health samples may be collected from different hosts and may also reflect different environmental materials and sites. Samples may be processed prior to library preparation and sequencing, and experiments may involve controls, replicates, as well as lab constructs (synthetic samples) for testing and optimization of methods and hypotheses. The Mpox specification provides a variety of fields for structuring sample collection and processing information. Scenarios and worked examples highlighting how different sample data structures can be used are provided below.

##### Harmonizing variable clinical sample descriptions

Examples of variable clinical sample descriptions harmonized using the Mpox specification are provided in Table 5. Original free text sample descriptions are provided paired with standardized terms sourced from field picklists to avoid lab-specific language use, abbreviations and to correct errors.

**Table 5.** Harmonized sample descriptions Original free text descriptions of clinical samples shown contain abbreviations, different vocabulary, different levels of granularity and cover a range of specimens, biomaterials and collection devices. The Mpox specification was used to clean and standardize the descriptions using different sample attributes. The cleaned data can be more easily used for identifying trends across clinical cases.

Original sample description	Anatomical material	Anatomical part	Collection device	Collection method	Biomaterial extracted
Anal dry swab		Anus [UBERON:0001245]	Dry swab [GENEPIO:0100493]		
Arm legion-pustule	Lesion (pustule) [NCIT:C78582]	Arm [UBERON:0001460]			
Crusted skin lesion perineal	Lesion (scab) [GENEPIO:0100490]	Perineum [UBERON:0002356]			
Superpubic skin crust	Lesion (scab) [GENEPIO:0100490]	Hypogastrium (suprapubic region) [UBERON:0013203]			
Genital swab		Genital area [BTO:0003358]	Swab [GENEPIO:0100027]		
Swab penis UTM		Penis [UBERON:0000989]	Swab [GENEPIO:0100027]; universal transport medium (UTM) [GENEPIO:0100509]		
Left knee lesion – leaky	Lesion (pustule) [NCIT:C78582]	Knee [UBERON:0001465]			
NP swab		Nasopharynx [UBERON:0001728]	Swab [GENEPIO:0100027]		
NP		Nasopharynx [UBERON:0001728]			
PLASMA	Blood plasma [UBERON:0001969]				
WHBLOOD	Whole blood [NCIT:C41067]				
CSF	Fluid (cerebrospinal (CSF)) [UBERON:0001359]				
Gargarism				Saline gargle (mouth rinse and gargle) [GENEPIO:0100034]	
DNA					DNA [CHEBI:16991]

##### Harmonizing specimen processing and linked sampling information

Samples may require processing prior to library preparation, and sometimes sampling events may be linked over time (e.g. cases of re-emergence or reinfection) or different samples may be collected from different anatomical parts/materials from the same individual. Processing of MPXV specimens may include activities such as virus passage and pooling of samples, among others. Specimen processing descriptions can be captured using the ‘specimen processing’ field, which provides a picklist of processes, as well as the ‘specimen processing details’ field, which enables the addition of free text notes. Samples may be linked to the same individual in different ways via the ‘host subject ID’ field. Scenarios and worked examples highlighting how specimen processing and sample linkage fields can be used are provided below. See Supplementary Material for additional examples.

**Worked examples**

Scenario 1: Multiple swabs pooled from different penile lesions (five swabs per sample), from patient (ABC12345), 14 August 2022.

**sample_collection_date:** 2022-08-14

**anatomical_material:** Lesion [NCIT:C3824]

**anatomical_part:** Penis [UBERON:0000989]

**collection_device:** Swab [GENEPIO:0100027]

**specimen_processing:** Specimens pooled [OBI:0600016]

**specimen_processing_details:** five swabs per sample

**host_subject_ID:** ABC12345

##### One health samples

One Health sampling strategies are often employed to better understand reservoirs of zoonotic disease, the risks of fomite transmission and other mechanisms of exposure. Scenarios and worked examples highlighting how different sample collection fields can be used to structure One Health sample descriptions are provided below.

**Worked examples**

Scenario 1: Wastewater was collected for MPXV surveillance.

**environmental_material:** Wastewater [ENVO:00002001]

Scenario 2: A sample of rat faeces was collected for MPXV surveillance.

**host_(common name):** Rat [NCBITaxon:10116]

**body_product:** Feces [UBERON:0001988]

Scenario 3: Bed linens in a group home were tested for the presence of MPXV.

**environmental_material:** Bedding (bed linen) [GSSO:005304]

**environmental_site**: Group home [ENVO:03501196]

### Host-associated information

Host data can include host scientific and common names, host age and gender, host health state and outcome data, clinical information (symptoms, pre-existing conditions and complications), exposure, treatment, prior infection and vaccination information. Scenarios and worked examples highlighting how host information fields can be used are provided below. See Supplementary Material for additional examples.

**Worked examples**

Scenario 1: A 21-year-old male who recently travelled to Toronto, Canada, was tested for Mpox upon exhibiting vesicular lesions after attending a party at which confirmed Mpox cases were present.

**host_(scientific name):** Homo sapiens [NCBITaxon:9606]

**host_(common name):** Human [NCBITaxon:9606]

**host_age**: 21

**host_age_unit:** Year [UO:0000036]

**host_gender:** Male [NCIT:C46109]

**host_health_state:** Symptomatic [NCIT:C25269]

**signs_and_symptoms:** Lesion (esicle) [GENEPIO:0100491] **destination_of_most_recent_travel_(city):** Toronto **destination_of_most_recent_travel_(country):** Canada [GAZ:00002560]

**exposure_event:** Party [PCO:0000035]

### Experimental design

Knowledge of sampling and sequencing strategies as well as other aspects of experimental design such as the inclusion of controls, replicates and synthetic data/lab constructs, can be critical for providing context when interpreting genomic surveillance data. In many fields, multi-tagging is possible if more than one standardized term applies. Fields for including free text notes are also available (e.g. ‘purpose of sequencing details’). Scenarios and worked examples highlighting useful experimental design fields are provided below. See Supplementary Material for additional examples.

**Worked examples**

Scenario 1: A sample originally collected for diagnostic purposes was retrospectively sequenced as part of a re-infection surveillance initiative. A note describing the criteria for sequencing selection was included.

**purpose_of_sampling:** Diagnostic testing [GENEPIO:0100002]

**purpose_of_sequencing:** Re-infection surveillance [GENEPIO:0100010]; Retrospective sequencing [GENEPIO:0100356

**purpose_of_sequencing_details:** samples selected from hospital records indicating a history of multiple infections

### Experimental methods (sequencing, bioinformatics and diagnostic testing)

Sequencing and bioinformatic methods can impact analytical results. Documenting these methods is critical for troubleshooting, as well as optimization, validation, reproducibility and auditability. The Mpox specification provides standardized fields and terms fields pertaining to sequencing assay types, library preparation, library enrichment strategies, sequencing protocols, insert/amplicon sizes, raw sequencing data file names and flow cell versions, as well as bioinformatics and quality control fields covering quality control methods and outcomes, bioinformatic processing, tool and database names and version numbers, protocols and commonly used metrics. Scenarios and worked examples highlighting different methods, fields and picklist terms are provided below.

**Worked examples**

Scenario 1: A sample that tested positive for the presence of MPXV (Ct value 34) was sequenced using a hybrid-capture enrichment approach (Illumina VSP panel library prep kit). The sample was sequenced using an Illumina NextSeq 2000 instrument on an S4 flow cell. FASTQ files (MPXV80_S1_L001_R1_001.fastq.gz, MPXV80_S1_L001_R2_001.fastq.gz) were stored in the laboratory’s data management system. Quality control of the sequence data was performed using an in-house pipeline called MPXV_QC v. 2.1.3, which flagged a low signal-to-noise ratio.

**sequencing_assay_type:** Whole virome sequencing assay [OBI:0002768]

**genomic_target_enrichment_method:** Hybridization capture [GENEPIO:0001950]

**sequencing_instrument:** Illumina NextSeq 2000 [GENEPIO:0100129]

**library_preparation_kit:** Illumina VSP panel

**sequencing_flow_cell_version:** S4

**r1_fastq_filename:** MPXV80_S1_L001_R1_001.fastq.gz

**r2_fastq_filename:** MPXV80_S1_L001_R2_001.fastq.gz

**quality_control_method_name:** MPXV_QC

**quality_control_method_version:** 2.1.3

**quality_control_determination:** Sequence flagged for potential quality control issues [GENEPIO:0100566]

**quality_control_issues:** Low signal to noise ratio [GENEPIO:0100574]

**quality_control_details:** CT value of 34. Low viral load. Low DNA concentration after amplification.

Scenario 2: MPXV sequence data was generated using an Oxford Nanopore sequencing instrument (total reads 423,867). The virus’ taxonomic identity was determined by aligning the reads to the EPI2ME reference database using the EPI2ME MPXV software package (v. 24.08-01). A taxonomic report was generated (MPXV_report186b.doc) on 24 September 2024. The complete bioocol was described in a lab GitHub repository (https://github.com/VirLab/mpxv).

**number_of_total_reads:** 423,867

**read_mapping_software_name:** EPI2ME

**read_mapping_software_version:** 24.08-01

**taxonomic_reference_database_name:** EPI2ME

**taxonomic_reference_database_version:** 24.08-01

**taxonomic_analysis_report_filename:** MPXV_report186b.doc

**taxonomic_analysis_date:** 2024-09-24

**bioinformatics_protocol:** https://github.com/VirLab/mpxv

### Integrated worked example

An integrated example is provided in order to illustrate what a complete contextual data record might look like. The example is extensive in order to showcase a wide variety of fields and terms; however, it should be noted that only the fields that are pertinent to a laboratory’s needs and objectives need to be filled. The amount of information captured is at the discretion of the data steward, and labs are under no obligation to share data.

Scenario: A 20-year-old unvaccinated male recently attended a party during which he engaged in sex with an individual who was later confirmed to be infected with MPXV. The male began exhibiting signs of fever, rash and swollen lymph nodes and eventually lesions. This individual attended a clinic (Centre de Santé Médicale Bellevue, France; contact for follow-up: bloggs@csmb.fr) and macule lesions on his back were swabbed on 18 August 2024 for diagnostic purposes. He was instructed to self-quarantine at home. The sample (AABC199) was later sequenced using the Yale tiling amplicon scheme and an Illumina MiSeq, on 26 August 2024 by the Laboratoire du Centre de Santé Médical Griffiths. Sequencing was performed as part of a priority surveillance project in the men who have sex with men (MSM) community. The virus being sequenced was assigned an identifier (described as isolate MPXV/human/FRA/AABC199/2024). A consensus sequence was generated using the ARTIC pipeline described at https://github.com/artic-network/artic-mpxv-illumina-nf which includes read trimming and adapter removal, dehosting, read mapping and alignment to the reference sequence.

**specimen_collector_sample_ID:** AABC199

**sample_collected_by:** Centre de Santé Médicale Bellevue

**sample_collector_contact_email:** bloggs@csmb.fr

**sample_collection_date:** 2024-08-18

**geo_loc_name_(country):** France [GAZ:00003940]

**geo_loc_name_(state/province/territory):** Not Provided [GENEPIO:0001619]

**organism:** Mpox virus [NCBITaxon:10244]

**isolate:** MPXV/human/FRA/AABC199/2024

**purpose_of_sampling:** Diagnostic testing [GENEPIO:0100002]

**anatomical_material:** Lesion (Macule) [NCIT:C43278]

**anatomical_part:** Back [FMA:14181]

**collection device:** Swab [GENEPIO:0100027]

**host_(common name):** Human [NCBITaxon:9606]

**host_(scientific name):** Homo sapiens (NCBITaxon:9606)

**host_health_state:** Symptomatic [NCIT:C25269]

**host_health_status_details:** Self-quarantining [NCIT:C173768]

**host_disease:** Mpox [MONDO:0002594]

**host_age:** 20

**host_age_unit:** Year [UO:0000036]

**host_age_bin:** 20–29 [GENEPIO:0100051]

**host_gender:** Male [NCIT:C46109]

**signs_and_symptoms:** Fever [HP:0001945]; Rash [HP:0000988]; Swollen lymph nodes [HP:0002716]

**host_vaccination_status:** Not vaccinated [GENEPIO:0100102]

**exposure_event:** Party [PCO:0000035]

**exposure_contact_level:** Sexual transmission [NCIT:C19085]

**host_role:** Sexual partner of case [GENEPIO:0100500]

**sequenced_by:** Laboratoire du Centre de Santé Médical Griffiths

**sequence_submitted_by:** Not Applicable [GENEPIO:0001619]

**purpose_of_sequencing:** Targeted surveillance (Non-random sampling) [GENEPIO:0100006]

**purpose_of_sequencing_details:** MSM surveillance priority

**sequencing_assay_type:** Amplicon sequencing assay [OBI:0002767]

**sequencing_date:** 2024-08-26

**sequencing_instrument:** Illumina MiSeq [OBI:0002003]

**amplicon_pcr_primer_scheme:** Yale

**raw_sequence_data_processing_method:** artic-mpxv-illumina-nf

**dehosting_method:** artic-mpxv-illumina-nf

**consensus_sequence_software_name:** artic-mpxv-illumina-nf

**consensus_sequence_software_version:** 1.0.0

### Data curation

One of the most valuable lessons learned in the implementation of the Mpox specification – as well as other related contextual data standards – is that while many data entry, validation and transformation functions can be automated, manual data curation will always be critical for efficient pathogen genomics data harmonization. Canadian Mpox genomic surveillance data submitted to the national genomics database at the Public Health Agency of Canada’s National Microbiology Laboratory was structured and curated by data generators (national and provincial public health labs) and reviewed by dedicated personnel to ensure compliance with the Mpox contextual data standard. While most errors could be detected using the DataHarmonizer validation, some data providers preferred to populate data programmatically and relied on in-house scripting. Manual review of submitted datasets was able to catch scripting errors, as well as other errors that could not be identified in an automated fashion by the DataHarmonizer, e.g. copy and paste errors from Excel files such as dates that increment chronologically down columns (correct format, but unlikely pattern). Manual curation was also helpful when information was included in records as free text in ‘details’ fields, e.g. host exposure information recorded in ‘purpose of sequencing details’ when the information could be more appropriately captured in other fields such as ‘exposure setting’ and ‘exposure event’. Improvements and errors detected by manual curation were treated as lessons learned, which were then used to iteratively improve the curation protocol.

Sequence data and accompanying contextual data was publicly shared via submissions to NCBI. Contextual data was formatted using the ‘Pathogen: clinical or host-associated’ metadata package (Pathogen.cl). As edits and updates sometimes occurred directly to data already entered into the PHAC-NML’s national genomic database, data retrieved from the national database was also reviewed prior to upload to NCBI. As the ‘isolation source’ field is used in many downstream pipelines at NCBI (e.g. pathogen detection), sample descriptors from different fields in the Mpox specification were concatenated and included in the ‘isolation source’ field in NCBI records. Manual curation checks helped to ensure consistency of automated transformations, which was key when a sample represented an edge case. Well-trained curators, knowledgeable about how to use data management tools as well as adept at communicating between data standards developers, data providers and software developers, are integral to the data management ecosystem but are sadly often left out of the operational and budgetary planning. Recommendations regarding data curation developed from experience and many lessons learned are provided in Table 6. Furthermore, curation of Mpox and other genomic surveillance contextual data contributed to the semantic best practices for improving standardized terms, interoperability and data quality.

**Table 6.** Curation recommendations for curators, data providers, software developers and decision-makers based on lessons learned from different pathogen surveillance initiatives

Target audience	Recommendation
**Curators**	If information (e.g. sample description) is unclear or abbreviations are used, it is best to go back to the data provider for clarification before integrating into databases or propagating the information to downstream repositories; if the meaning of information is unclear to the curator, it will likely be unclear to the rest of the community; avoid forcing free text descriptions to fit into existing picklist terms; if the appropriate terms appear to be unavailable, consult the standard developer for further information and/or new term requests; document the problems you solve and the issues that arise so these can be raised with data providers, standards developers and software developers and so that decision-makers are aware of your contributions; communicate data needs to data standards developers as well as tool developers
**Data providers**	Avoid abbreviations in contextual data; provide contact information and use consistent organizational names in records (e.g. same spelling); as much as possible, dedicate trained staff for data curation as clean data is critical for analyses and for data sharing when mistakes can be propagated and amplified
**Software developers**	Design contextual data collection instruments based on data standards; design contextual data management systems based on ontology identifiers rather than the vocabulary labels as these can vary (e.g. synonyms or multilingual translations); humans have politics and preferences, but computers do not
**Decision-makers**	Genomic data provides value if it can be easily used and reused; invest in the longevity of data by future-proofing it through the use of data standards; dedicate funding for data management and curation (including systems and standards development and maintenance, personnel and training)

## Discussion

Rich, well-structured contextual data is critical for pathogen surveillance, public health decision-making and research and innovation. This work describes the development and implementation of a contextual data specification for Mpox virus genomic surveillance for public health action. The specification was designed using an ISO-based framework, open source ontologies and semantic best practices for improving machine readability. The Mpox specification is part of a growing library of interoperable genomics surveillance contextual data standards. While the Mpox specification can be used to harmonize a wide variety of One Health contextual data, we recommend that strictly wastewater-based detection of MPXV be captured and harmonized using a new Wastewater Contextual Data Specification developed by the Public Health Alliance for Genomic Epidemiology (https://github.com/pha4ge/Wastewater_Contextual_Data_Specification). Templates based on the PHA4GE Wastewater Specification are also provided in the DataHarmonizer in the ‘pathogen-genomics-package’ (https://github.com/cidgoh/pathogen-genomics-package). Mpox contextual data from wastewater samples is best captured using the Wastewater Pathogen Agnostic template, which provides a module for Environmental Measurement and Conditions, and contains a wide range of physico-chemical attributes (e.g. flow rates, dissolved solids, pH and temperature). The Mpox and wastewater specifications are interoperable, better enabling integration of clinical and environmental surveillance data. Both standards are scoped for sequencing strategies based on tiling amplicon, hybrid-capture enrichment and metagenomic approaches. Although development turnaround times vary depending on data complexity, reuse of existing modules helped to speed up the development of both specifications. Both of these data standards were recently included in a WHO Collaboratory Community of Practice resource hub (https://www.who.int/initiatives/collaboratory).

It should be noted that the specification is intended to facilitate harmonization of data at different levels, i.e. to facilitate streamlined data capture within local public health databases for internal use, to enable sharing with trusted partners/within networks and to facilitate public sharing. As public health priorities are different across regions and communities, the standard provides flexibility in the range of information that can be captured. The reference guides and curation SOP provide guidance on granularity and re-identification risks for different kinds of data. However, how the parts of the entire specification are used and whether and how data is shared are up to individual public health organizations.

With the increase in routine use of genomic surveillance, there are increasing demands for data standards from the community [36]. Standard development requires experience and expertise. Despite the increase in development efficiency provided by the reuse of existing modules, fields and terms, scaling up of standards development continues to be challenging due to the lack of formalized training. This situation is analogous to the need for formalized bioinformatics training programmes which began over a decade ago. Training programmes like the Bioinformatics.ca Infectious Disease Genomic Epidemiology Workshop (https://bioinformatics.ca/workshops-all/2024-infectious-disease-genomic-epidemiology-virtual/) have started to incorporate training in data curation and the implementation of data standards. CIDGOH has also partnered with PHA4GE to develop data standards development training materials (e.g. https://github.com/cidgoh/specification-development-training). PHA4GE is also working with the WHO’s International Pathogen Surveillance Network to develop a Public Health Genomics Data Standards Catalogue to help centralize interoperable community standards developed globally and to establish training manuals and quality criteria. Owing to the close relationship between CIDGOH and PHA4GE, the Catalogue’s inclusion criteria will be informed by the best practices described in this work. Both organizations also encourage the sharing of standard-based data collection templates.

There is an ongoing need for new data management tools. Based on community feedback and testing, CIDGOH is currently working to improve the DataHarmonizer by enabling multilingual functionality as well as the ability to customize templates based on existing specifications. This new functionality will enable organizations to mix-and-match fields and terms according to their own data needs while adhering to global standards and best practices. However, the DataHarmonizer is not, and should not be, the only solution. We encourage the community to respond to unmet contextual data quality control needs by integrating data standards into new curation and analytical tools, as well as lab information management systems and repositories. This call to action is already being answered by Pathoplexus and VIRUS-MVP, which integrate ontology-based data structures into their schemas. Pathoplexus offers flexible data sharing options enabling users to share their data openly or with time-limited protections to ensure proper attribution and safeguard their contributions. After a specified period, Pathoplexus facilitates upload to INSDC-member databases helping to make data openly accessible and minimizing barriers for data providers. Recently, Pathoplexus has added Mpox to the repertoire of pathogens it supports. VIRUS-MVP is a heatmap-centric visualization web application that encodes mutational information across multiple groups, including SARS-CoV-2 and, recently, MPXV lineages [37].

The Mpox specification package presented in this work is providing much needed interoperability and helps to align processes of data collection, storage, sharing and analysis. This package, and the growing library of compatible standards, is already improving data flows across the global data ecosystem.

## Supplementary material

10.1099/mgen.0.001614 Uncited Supplementary Material 1.

## Abbreviations

CIDGOH: centre for infectious disease genomics and one health
COVID-19: Coronavirus disease 2019
ENA: european nucleotide archive
GISAID: global initiative on sharing avian influenza data
INSDC: international nucleotide sequence database collaboration
IPSN: international pathogen surveillance network
MPXV: Mpox virus
MSM: men who have sex with men
NCBI: national center for biotechnology information
OBO: open biological and biomedical ontology
SARS-CoV-2: severe acute respiratory syndrome coronavirus 2
WHO: world health organization
