# Supplementary material for: The Mpox contextual data specification package: a data curation toolkit to support collaborative pathogen genomic surveillance
Source: Microb Genom. 2026 Jan 23;12(1):001614. doi: 10.1099/mgen.0.001614 (PMC13293314; doi:10.1099/mgen.0.001614)
Supplement: Uncited Supplementary Material 1. [file mgen-12-01614-s001.pdf]

Supplementary Table 1 - Genbank Sequence Accessions for Mpox Consensus Sequences

Genbank Accession
OP279049.1
OP279048.1
OP279047.1
OP279046.1
OP279045.1
OP279044.1
OP279043.1
OP279042.1
OP279041.1
OP279040.1
OP279039.1
OP279038.1
OP279037.1
OP279036.1
OP279035.1
OP279034.1
OP279033.1
OP270029.1
OP270028.1
OP270027.1
OP270026.1
OP270025.1
OP270024.1
OP270023.1
OP270022.1
OP270021.1
OP226139.1
ON880519.3
OP062236.1
OP062235.1
OP062234.1
OP062233.1
OP062232.1
OP062231.1
OP062230.1
OP062229.1
OP013017.1
OP013016.1
OP013015.1
OP013014.1
OP013013.1
OP013012.1

OP013011.1
OP013010.1
OP013009.1
OP013008.1
OP013007.1
OP013006.1
OP013005.1
OP013004.1
OP013003.1
OP013002.1
OP013001.1
OP983167.1
OP983166.1
OP983165.1
OP983164.1
OP983163.1
OP983162.1
OP983161.1
OP983160.1
OP983159.1
ON880549.1
ON880548.1
ON880547.1
ON880546.1
ON880545.1
ON880544.1
ON880543.1
ON880542.1
ON880541.1
ON880540.1
ON880539.1
ON880538.1
ON880537.1
ON880536.1
ON880535.1
ON880534.1
ON880533.1
ON880532.1
ON880531.1
ON880530.1
ON880529.1
ON880528.1
ON880527.1
ON880526.1
ON880525.1
ON880524.1
ON880523.1
ON880522.1
ON880521.1
ON880520.1

ON880518.1
ON880517.1
ON880516.1
ON880515.1
ON880514.1
ON880513.1
ON880512.1
ON880511.1
ON880510.1
ON880509.1
ON880508.1
ON880507.1
ON880506.1
ON880505.1
ON803444.1
ON803443.1
ON803442.1
ON803441.1
ON803440.1
ON803439.1
ON803438.1
ON803437.1
ON803436.1
ON803435.1
ON803434.1
ON803433.1
ON803432.1
ON803431.1
ON803430.1
ON803429.1
ON803428.1
ON803427.1
ON803426.1
ON803425.1
ON803424.1
ON803423.1
ON803422.1
ON803421.1
ON803420.1
ON803419.1
ON803418.1
ON803417.1
ON803416.1
ON803415.1
ON803414.1
ON803413.1

Supplementary Additional Worked Examples

Identifier Tracking

Scenario: A sample was collected in a clinic and assigned an identifier (XYZ999-c). The sample was obtained from a patient that was assigned an anonymized identifier in the clinic's lab information management system to protect their privacy (ABC12345). The patient was suspected to be a case involved in an outbreak and the current episode of infection was assigned a case ID (X00-AB133). The sample was sent for sequencing at the national reference laboratory where it was processed. As sequencing protocols were undergoing optimization, the library prep was assigned an identifier by the sequencing lab (LP2004-2024). Once sequencing was complete, the raw sequence data and accompanying contextual data was submitted to NCBI (BioProject PRJNA12345; BioSample SAMN14180202; SRA accession SRR123456). A partial contextual data record for the sample highlighting different identifier tracking fields, is provided below.

specimen_collector_sample_ID: XYZ999-c
host_subject_ID: ABC12345
case_ID: X00-AB133
library_ID: LP2004-2024
BioProject_accession: PRJNA12345
BioSample_accession: SAMN14180202
INSDC_sequence_read_accession: SRR123456

Longitudinal Sampling

Scenario: Swabs of groin lesions from patient (#ABC12345), 2 weeks after initial sampling (August 28 2022)

sample_collection_date: 2022-08-28
purpose_of_sequencing: Longitudinal surveillance (repeat sampling of individuals) [GENEPIO:0100009]
anatomical_material: Lesion [NCIT:C3824]
anatomical_part: Genital area [BTO:0003358]
collection_device: Swab [GENEPIO:0100027]
host_subject_ID: ABC12345

Vaccination

Scenario: A partially vaccinated male (one dose JYNNEOS vaccine; requires 2 doses for full vaccination) was sampled as part of a vaccination surveillance program.
host_gender: Male [NCIT:C46109]
host_vaccination_status: Partially Vaccinated [GENEPIO:0100101]
number_of_vaccine_doses_received: 1
vaccination_dose_1_vaccine_name: JYNNEOS vaccine

Replicate Tracking

Scenario 2: A lab is optimizing their sequencing protocols. A specimen (ABCD789) was subsampled into two technical replicates. A library (ABCD789-i) was prepared using one of the replicates using protocol 1A. The sequence was tagged as a replicate using the "experimental_specimen_role_type" field.

specimen_collector_sample_ID: ABCD789
experimental_specimen_role_type: Technical replicate [EFO:0002090]
library_ID: ABCD789-i
sequencing_protocol: 1A

PCR Test Methodology and Results

Fields are also provided for capturing qPCR data which is useful for triaging samples for sequencing and comparing viral loads. Unified Orthopoxvirus gene names are used in the specification to refer to targets, however, Expasy Viral Zone mappings to Vaccinia Copenhagen nomenclature are included as synonyms in ontology annotations (Viral Zone, 2024).

Scenario 3: A sample was tested for the presence of the Mpox virus using a diagnostic qPCR assay named B6R described by Liu et al, 2006. The assay was based on the Mpox orthopoxvirus gene 190 (opg190) which encodes an EEV type-I membrane glycoprotein. The test produced a Ct value of 26.

gene_symbol_1: opg190
diagnostic_pcr_protocol_1: B6R (Liu et al, 2006)
diagnostic_pcr_Ct_value_1: 26

Taxonomy

Mpox virus taxonomy can be determined via different molecular and sequence-based methods. Taxonomy of viral samples can be captured using different fields in the specification, which include the "organism" field which can be used to identify the pathogen as well as different lineage/clade fields. The "lineage/clade_name" field provides a picklist of different Mpox I and II clades and subclades. As the standard is reviewed periodically, taxonomy conventions are updated. A worked example highlighting the use of these fields is provided below.
Scenario: A sample from a patient that tested positive by qPCR for Mpox is sequenced and compared to other viruses circulating in the community. The sample is determined to contain Mpox virus Clade Ia using IQ-Tree v 2.3.6, as part of the Nextstrain software package.

organism: Mpox virus [NCBITaxon:10244]
lineage/clade_name: Mpox virus clade Ia [GENEPIO:0102030]

lineage/clade_analysis_software_name: IQ-TREE
lineage/clade_analysis_software_version: 2.3.6
